# Synthesis of Highly Photoluminescent All-Inorganic CsPbX_3_ Nanocrystals via Interfacial Anion Exchange Reactions

**DOI:** 10.3390/nano9091296

**Published:** 2019-09-11

**Authors:** Zongtao Li, Cunjiang Song, Longshi Rao, Hanguang Lu, Caiman Yan, Kai Cao, Xinrui Ding, Binhai Yu, Yong Tang

**Affiliations:** 1 Engineering Research Centre of Green Manufacturing for Energy-Saving and New-Energy Technology, School of Mechanical and Automotive Engineering, South China University of Technology, Guangzhou 510640, China; meztli@scut.edu.cn (Z.L.); guyuexuan1999@foxmail.com (H.L.); chamenyan@163.com (C.Y.); 201821002410@mail.scut.edu.cn (K.C.); dingxr@scut.edu.cn (X.D.); bhaiyu@yeah.net (B.Y.); ytang@scut.edu.cn (Y.T.); 2 College of Engineering, Shantou University, Shantou 515063, China

**Keywords:** Cs_4_PbBr_6_ NCs, CsPbX_3_ NCs, interfacial anion exchange, chemical transformation

## Abstract

All-inorganic cesium lead halide perovskite CsPbX_3_ (X = Cl, Br, I) nanocrystals (NCs) have attracted significant attention owing to their fascinating electronic and optical properties. However, researchers still face challenges to achieve highly stable and photoluminescent CsPbX_3_ NCs at room temperature by the direct-synthesis method. Herein, we synthesize CsPbX_3_ NCs by a facile and environmentally friendly method, which uses an aqueous solution of metal halides to react with Cs_4_PbBr_6_ NCs via interfacial anion exchange reactions and without applying any pretreatment. This method produces monodisperse and air-stable CsPbX_3_ NCs with tunable spectra covering the entire visible range, narrow photoluminescence emission bandwidth, and high photoluminescence quantum yield (PL QY, 80%). In addition, the chemical transformation mechanism between Cs_4_PbBr_6_ NCs and CsPbX_3_ NCs was investigated. The Cs_4_PbBr_6_ NCs were converted to CsPbBr_3_ NCs first by stripping CsBr, and then, the as-prepared CsPbBr_3_ NCs reacted with metal halides to form CsPbX_3_ NCs. This work takes advantage of the chemical transformation mechanism of Cs_4_PbBr_6_ NCs and provides an efficient and environmentally friendly way to synthesize CsPbX_3_ NCs.

## 1. Introduction

In recent years, all-inorganic cesium lead halide perovskite CsPbX_3_ (X = Cl, Br, and I) nanocrystals (NCs) have attracted a lot of attention due to their excellent properties, such as high photoluminescence (PL) quantum yield (QY), broad wavelength coverage, narrow PL emission bandwidth, and low trap state density [1,2,3,4]. These outstanding properties of CsPbX_3_ NCs distinguish them from traditional semiconductor NCs and make them promising candidates for various optoelectronic applications, including solar cells [5], lasers [6], photodetectors [7], and light emitting diodes (LEDs) [8].

To date, many methods have been developed for the synthesis of CsPbX_3_ NCs, such as hot-injection [9], solvothermal synthesis [10], post-treatment [11], ultrasonication [12], and mechanochemistry [13,14]. However, researchers still face challenges to achieve highly stable and photoluminescent CsPbX_3_ NCs at room temperature by the direct-synthesis method. In addition to studying various direct synthesis methods, the chemical transformation of pre-synthesized NCs has drawn researchers’ attention in synthesizing CsPbX_3_ NCs because they allow for the precise control of the NCs and enable the synthesis of materials with different morphologies and sizes, such as nanowires and nanorods that are difficult to obtain directly [15,16]. Recently, the chemical transformation between non-luminescent Cs_4_PbX_6_ and luminescent CsPbX_3_ has sparked researchers’ interest. For example, the Akkerman group [17] treated pre-synthesized Cs_4_PbX_6_ NCs with excess PbX_2_ to synthesize green fluorescent CsPbX_3_ NCs. The Jing group [18] reported highly luminescent CsPbBr_3_ nanorods (NRs) through chemical transformation from Cs_4_PbBr_6_ NCs and tuned PL emission of the NRs over the full visible range through a halide anion-exchange reaction using hot-injection. During these processes, anion exchange is a common strategy used to modify the chemical composition of as-prepared NCs and regulate their spectrum. However, most chemical transformation anion exchange processes use oleylamine-X or PbX_2_ as anion sources, which require inert conditions, high temperatures, and pre-treated precursors [19]. For example, anion sources, such as PbX_2_ need to be dissolved in octadecene (ODE) first and then a is added, such as oleic acid or oleylamine, to dissolve the halogen ions. Moreover, in order to obtain highly stable and PL QY NCs, inert conditions are required during the anion exchange process. These anion exchange methods are limited to the direct mixing of solid halogen ions with perovskite solutions, which not only requires a cumbersome dissolution process but also brings in a large number of non-luminescence materials during the anion exchange process.

Ion transport across the interface between two immiscible liquids is a fundamental study in catalysis and electrochemistry, namely, different ions can be transferred through the interface of two incompatible solvent interfaces. Herein, we report a novel interfacial anion exchange route in the chemical transformation of Cs_4_PbBr_6_ to CsPbX_3_ on the n-hexane/water interface. Taking advantage of ion transport across the interface between two immiscible liquids, we can easily synthesize highly stable and PL QY CsPbX_3_ NCs at room temperature. By using different metal halides, the band-gap energy and PL spectra were readily tuned over the entire visible spectral region. Differing from the mostly reported chemical transformation used anion sources, the metal halides are dissolved in deionized water due to their high solubility in water. This process does not need any complicated pretreatment, such as heating in a vacuum or adding oleic acid and oleylamine to dissolve the halide source, which not only improves synthetic efficiency and product purity but also lowers the production cost. In addition, the chemical transformation mechanism between Cs_4_PbBr_6_ NCs and CsPbX_3_ NCs was investigated. We further demonstrated the potential applications of the perovskite NCs in white LED (WLEDs) by combining the green luminescent CsPbBr_3_ NCs with red luminescent CdSe NCs and a blue GaN LED chip, showing an encompassing 125% color gamut of the National Television System Committee (NTSC, 1913) standard.

## 2. Materials and Methods

### 2.1. Chemicals and Materials 

Cesium carbonate (Cs_2_CO_3_, 99.99%), lead bromide (PbBr_2_, 99.99%), 1-octadecene (ODE, 90%), oleic acid (OA, 90%), and oleylamine (OAm, 90%) were purchased from Sigma-Aldrich Co. (St. Louis, MO, USA). N-hexane, methyl acetate, and poly (methyl methacrylate) (PMMA) were purchased from Shanghai Aladdin Biochemical Technology Co. (Shanghai, China). Transparent silicone resin (OE-6650A and B) was purchased from Dow Corning Co. (Midland, MI, USA). All chemicals were used as received without any further purification.

### 2.2. Preparation of Cesium-Oleate Solution

A total of 0.16 g Cs_2_CO_3_ (0.49 mmol), 1.0 mL OA and 16 mL ODE were loaded into a 30 mL flask, dried for 30 min at 120 °C under N_2_, and then heated to 150 °C until all Cs_2_CO_3_ reacted with OA to form a cesium-oleate solution.

### 2.3. Preparation of Cs_4_PbX_6_ NCs

In a typical synthesis of Cs_4_PbBr_6_ NCs, 1.0 mL OAm, 1.0 mL OA, 10 mL ODE, and 0.2 mmol PbBr_2_ were loaded into a 25 mL three-neck flask and dried under vacuum for 30 min. The reaction system was heated to 140 °C. Then, hot (~150 °C) Cs-oleate solution (4.4 mL in ODE, the molar ratio of Cs-oleate and PbBr_2_ was 1.35:1, prepared as described above) was rapidly injected into the PbBr_2_ solution. Seven seconds later, the mixture was immediately cooled with an ice-water bath.

### 2.4. Interfacial Anion Exchange Reactions

**Scheme 1:**
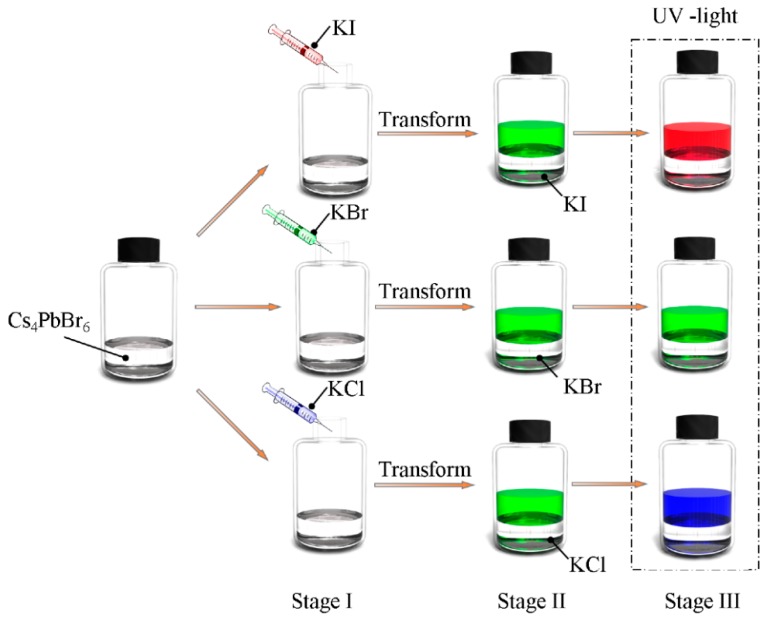
Schematic diagram of synthesizing CsPbX_3_ (X = Cl, Br, and I) by an interfacial anion exchange reaction. Stage I shows the color of Cs_4_PbBr_6_ solution before KX added; Stage II shows the color of Cs_4_PbBr_6_ solution changed to green within several seconds when KX was added; Stage III shows the color of Cs_4_PbBr_6_ solution changed to red when KI was added for 24 h and changed to blue-purple when KCl was added for 32 h, respectively.

The anion exchange process on the interface of n-hexane/KX aqueous solution is shown in Scheme 1. The reaction was conducted under ambient condition. In a typical interfacial anion exchange process to prepare CsPbI_3_, 3.0 mL Cs_4_PbBr_6_ n-hexane dispersion was added into a 10 mL glass bottle. The original Cs_4_PbBr_6_ solution is colorless, as shown in Scheme 1-Stage I. Then, 3.0 mL KI aqueous solution (0.5 M) was injected quickly, the color of the upper Cs_4_PbBr_6_ n-hexane dispersion was changed from colorless to green rapidly under UV light irradiation, as shown in Scheme 1-Stage II. After 24 h, the color of supernatant gradually turned yellow, orange, and eventually red, as shown in Scheme 1-Stage III. When KI aqueous solution was replaced by KCl aqueous solution, the upper green n-hexane dispersion would turn indigo and then blue-purple. This process takes 32 h or even more. In addition, when KI aqueous solution was substituted with KBr aqueous solution, the color of the upper green n-hexane dispersion remained unchanged. Moreover, slightly disturbing the mixing solution during this process would increase the anion exchange rate. The KX can be substituted by other metal halides, such as NaX, ZnX_2_, CuX_2_, MgX_2_, and CaX_2_, the color change of the mixture is almost as the same as KX, while the reaction time is variable depending on the halide source.

### 2.5. Fabrication of WLEDs Devices 

To obtain highly efficient WLEDs, a blue GaN chip with a rough structure was applied [20]. The WLEDs devices consist of a CsPbBr_3_ NC film, a CdSe NC film, and a blue GaN chip. To fabricate green CsPbBr_3_ NCs film, the as-synthesized CsPbBr_3_ NCs were dispersed into a PMMA/n-hexane solution and the mixture was vigorously stirred with a vacuum homogenizer for 6 min to degas. After that, the mixture was injected into a mold and heated at 60 °C for 2 h to remove n-hexane. The CdSe film was prepared by adding red CdSe NCs into the silicone gel (OE-6650 A and B) and vigorously stirring with a vacuum homogenizer for 12 min to degas. Then, the mixture was injected into a mold and solidified in a vacuum oven at 80 °C for 30 min and at 150 °C for 2 h, sequentially. Finally, the green and red film layers were orderly coated on the surface of a blue GaN chip.

### 2.6. Characterizations

The crystal phase and surface morphology of the samples were characterized by transmission electron microscopy (TEM, JEM-2100F, JEOL, Tokyo, Japan) with an accelerating voltage of 200 kV. The phase of the as-prepared perovskite NCs were measured using an X-ray diffractometer (XRD, D8-Advance, Bruker, Germany) with a Cu–Kα radiation source (*λ* = 0.15418 nm) at a counting rate of 2° per minute in the scanning angle (2*θ*) range from 5° to 50°. The ultraviolet-visible (UV-vis) absorption spectra of the samples were tested using a UV-vis spectrometer (Shimadzu, Kyoto, Japan) over the wavelength range from 300 nm to 700 nm, at 1 nm intervals. The photoluminescence (PL) spectra of the products were recorded using a fluorescence spectrophotometer (RF-6000, Shimadzu, Kyoto, Japan) with a Xe lamp as an excitation source.

## 3. Results and Discussion

**Figure 1:**
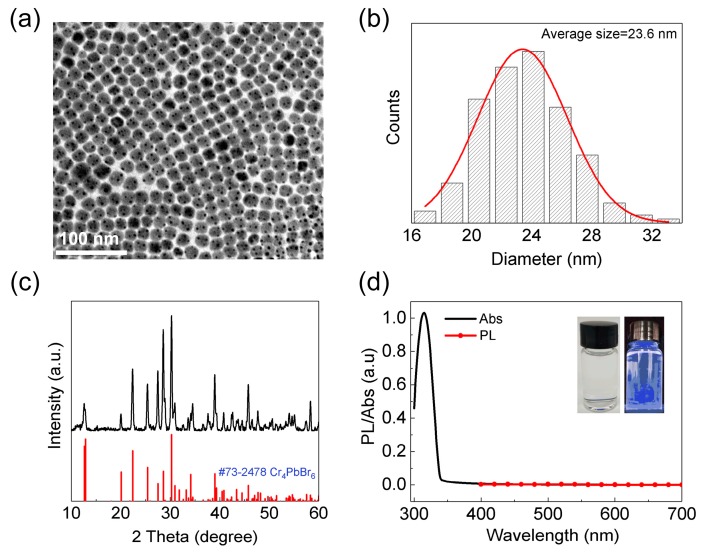
Characterization of Cs_4_PbBr_6_ nanocrystals (NCs): (**a**) TEM image, (**b**) particle size distribution, (**c**) X-ray diffractometer (XRD) pattern, and (**d**) UV-vis absorption and photoluminescence (PL) spectra. Insets are the photographs of Cs_4_PbBr_6_ NCs under sunlight and UV light irradiation.

The surface morphology of the as-synthesized Cs_4_PbBr_6_ NCs was characterized by TEM, as shown in Figure 1a. The TEM image demonstrates that the Cs_4_PbBr_6_ NCs have a diamond-like shape and show good dispersion. Figure 1b indicates that Cs_4_PbBr_6_ NCs have a relatively wide particle size distribution and their average size is 23.6 nm. To determine the phase structure, XRD was applied to measure the as-synthesized Cs_4_PbBr_6_ NCs. As shown in Figure 1c, the XRD patterns show that Cs_4_PbBr_6_ NCs can be indexed as rhombohedral phase (JCPDS card No. 73-2478). In addition, no other phases were observed, suggesting a high sample purity.

The UV-vis absorption spectra and PL emission spectra of the original Cs_4_PbBr_6_ NCs are shown in Figure 1d. The UV-vis absorption spectrum has a single and spiculate absorption peak at 319 nm. This absorption feature is consistent with that of bulk Cs_4_PbBr_6_, which has been confirmed to be the localized 6s_1/2_-6p_1/2_ transition within the isolated [PbBr_6_]^4−^ octahedra separated by Cs^+^ ions [21,22]. No PL emission was observed for Cs_4_PbBr_6_ NCs. According to previous reports, the purified Cs_4_PbBr_6_ NCs did not show obvious PL emission over the entire visible range owing to their large bandgap (*E*_g_ = 3.94 eV) [23,24,25], suggesting the as-prepared Cs_4_PbBr_6_ NCs in this work are highly purified.

**Figure 2:**
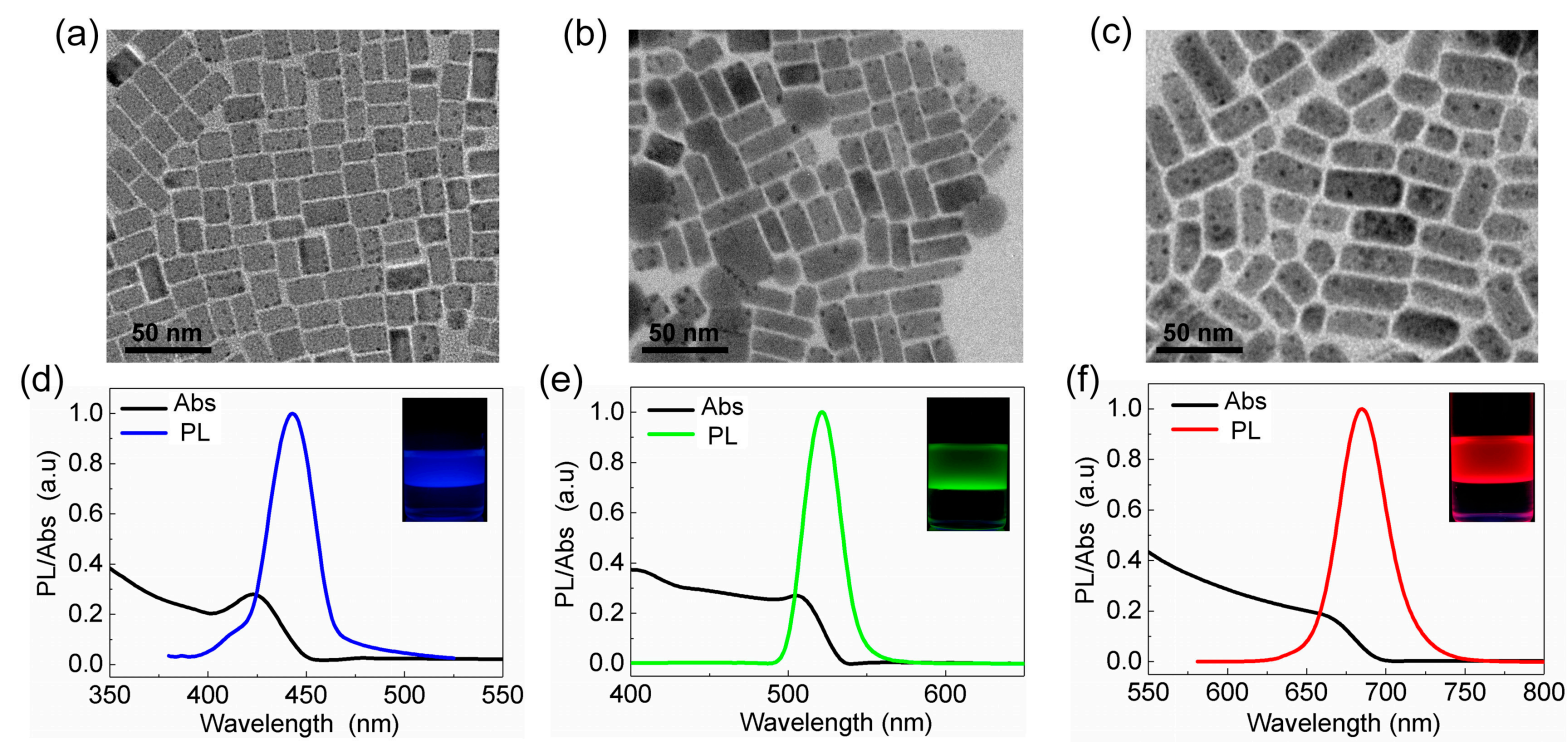
TEM images of samples prepared by Cs_4_PbBr_6_ n-hexane dispersion reaction with 0.5 mmol/mL KX (X = Cl, Br, I) aqueous solution: (**a**) KCl aqueous solution, (**b**) KBr aqueous solution, (**c**) KI aqueous solution. UV-vis absorption spectra and PL emission spectra of samples prepared by Cs_4_PbBr_6_ n-hexane dispersion reaction with 0.5 mmol/mL KX (X = Cl, Br, I) aqueous solution: (**d**) KCl aqueous solution, (**e**) KBr aqueous solution, (**f**) KI aqueous solution. The insets are digital photos of the products under UV light irradiation.

TEM was applied to characterize the three typical products obtained by interfacial anion exchange reactions. Figure 2a shows the TEM image of samples by adding KCl aqueous solution into Cs_4_PbBr_6_ n-hexane solution, demonstrating the samples have a regular cuboid shape with the average width of ca. 13 nm and length of ca. 32 nm, as shown in Figure S1a, b. When KCl was substituted with KBr or KI, the shape of products was almost the same as KCl, while the size and high-aspect-ratio are different, as shown in Figure 2b,c. For example, when the samples were directly obtained by Cs_4_PbBr_6_ transformation with KBr aqueous solution, the average width and length were 15 nm and 45 nm, respectively, and the high-aspect-ratio was 3. For the samples were synthesized by Cs_4_PbBr_6_ transformation with KI aqueous solution, the average width and length were 22 nm and 50 nm, respectively, and the high-aspect-ratio was 2.27. According to previous studies, this phenomenon is believed to be due to the self-assembly phenomenon of water on the formation of perovskite crystals [26,27,28]. This provides a new opportunity to directly synthesize crystals with different morphologies of other components. To determine the phase structure, XRD and high-angle annular dark field scanning TEM (HAADF-STEM) were used to characterize the as-prepared samples. The element mapping of HAADF-STEM in Figure S2 confirmed that the samples prepared by adding KCl into Cs_4_PbBr_6_ n-hexane solution were CsPbBr_1_Cl_2_. In addition, XRD patterns (Figure S3) indicated that the samples prepared using KBr and KI are CsPbBr_3_ and CsPbI_3_, respectively.

The UV-vis absorption and PL spectra of the corresponding samples are illustrated in Figure 2d,f. The CsPbBr_1_Cl_2_ NCs show a sharp first characteristic absorption at 427 nm and a strong PL emission peak at 435 nm with a full width at half maximum (FWHM) of 22 nm, as presented in Figure 2d. Furthermore, the small Stokes shift (8 nm) indicates the PL emission of CsPbBr_1_Cl_2_ NCs results from the bound exciton recombination [29]. In addition, the UV-vis absorption spectra and PL emission spectra of CsPbBr_3_ NCs show the first characteristic absorption peak of approximately 508 nm and the PL emission of around 520 nm with a narrow FWHM of 18 nm, and the UV-vis absorption peak and PL emission peak of CsPbI_3_ NCs were 670 nm and 690 nm, respectively. The PL QY of the as-prepared CsPbBr_1_Cl_2_ NCs, CsPbBr_3_ NCs and CsPbI_3_ NCs were measured to be ca. 45%, 80%, and 68% (Rhodamin 101 as reference, PL QY is 100%), respectively [30,31]. The insets in Figure 2d–f are representative digital pictures of each sample under 365 nm UV light irradiation, showing bright blue, green, and red fluorescence and indicating samples with high PL QY were formed. The above results clearly confirmed that the interfacial anion exchange processes were successfully completed for synthesizing different component products.

**Figure 3:**
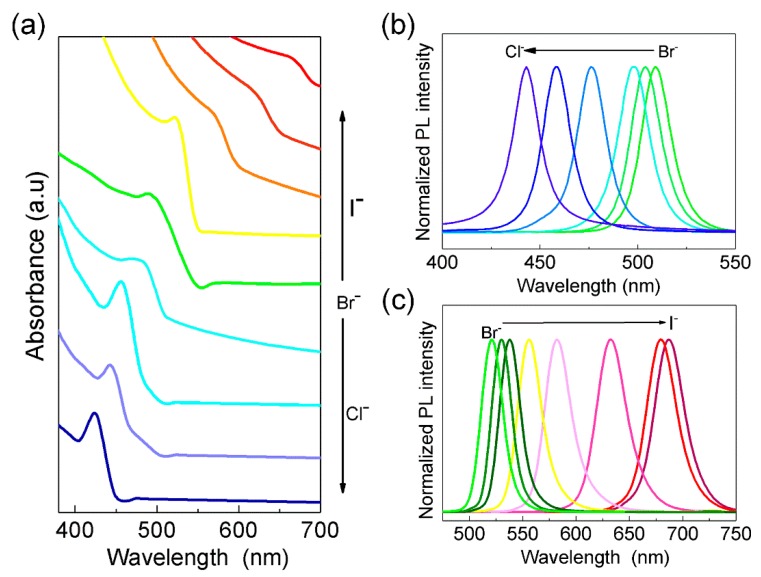
(**a**) Evolution of the UV-vis absorption spectra of samples that were prepared by the Cs_4_PbBr_6_ n-hexane dispersion reaction with 0.5 mmol/mL KX (X = Cl, Br, and I) aqueous solution. (**b**) PL spectra of the exchanged NCs obtained from the Cs_4_PbBr_6_ n-hexane dispersion by using (**b**) KCl and (**c**) KI aqueous solution as anion exchange resources.

To observe the spectral shift of these anion-exchange reactions in situ, representative UV-vis absorption spectra and PL spectra of such exchanged CsPbX_3_ NCs, are shown in Figure 3. By adding KCl into Cs_4_PbBr_6_ n-hexane solution, the first characteristic absorption peak of as-prepared samples gradually blue-shifted and finally reached 420 nm. This gradual shift is consistent with the XRD result due to the substitution of Br^−^ with Cl^−^. When KCl was substituted with KI, the absorption peak gradually red-shifted and finally reached 675 nm. In addition, we observed that the first characteristic absorption spectra gradually became sharper when adding KCl and became broader when adding KI over time, which is similar to a previously reported study [19]. The PL spectra of the CsPbX_3_ NCs that transformed from Cs_4_PbBr_6_ NCs are Stokes-shifted with respect to the optical absorption, as shown in Figure 3b,c. According to the interfacial ion exchange process, the UV-vis absorption and PL spectra matched well with those of the directly synthesized CsPbX_3_ NCs in previous reports [32], indicating that the CsPbX_3_ can be easily obtained by anion exchange in the interface of an n-hexane and KX aqueous solution.

**Figure 4:**
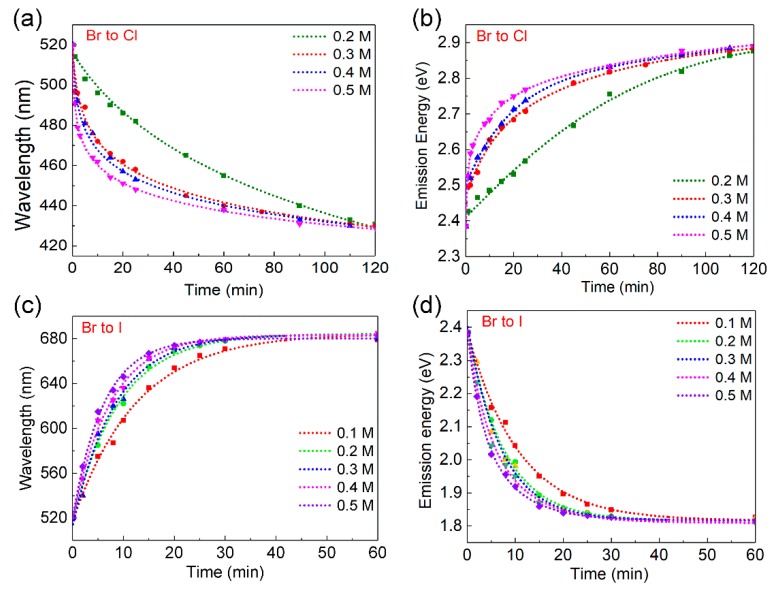
(**a**) PL wavelength and (**b**) emission peak energies of samples that were prepared by the Cs_4_PbBr_6_ n-hexane dispersion reaction with various concentrations (0.2 M ~ 0.5 M) of KCl aqueous solution. (**c**) PL wavelength and (**d**) emission peak energies of samples that were prepared by the Cs_4_PbBr_6_ n-hexane dispersion reaction with various concentrations (0.1 M ~ 0.5 M) of KI aqueous solution.

The concentration of KX plays an important role in the spectral shift during the anion exchange process. To study the effect of concentration of KX aqueous on optical properties of as-prepared samples, a series of KX aqueous concentrations control experiments have been conducted. During the interfacial anion exchange process, some aliquots of the reaction mixture were removed from the reaction system, and the samples were characterized by PL spectrometry. Figure 4a,b show the emission peak position and PL emission energy as a function of time after adding different concentrations of KCl solution into the Cs_4_PbBr_6_ n-hexane solution. When the concentration of KCl was 0.2 M, the PL emission peak increased from 520 nm to 425 nm over time, and the corresponding PL emission energy increased from 2.38 eV to 2.88 eV. As the concentration of KCl increased, the rate of blue-shift increased. This is because the increase of ion concentration results in the increase of the frequency of ion contact, which leads to the acceleration of the exchange rate. When KI was added to the Cs_4_PbBr_6_ n-hexane solution, the PL emission peak shifted from 520 nm to 650 nm with a displacement of 130 nm, and the corresponding emission energy decreased from 2.38 eV to 1.90 eV (Figure 4c,d). Increasing the concentration of KX will improve the exchange rate, but the ultimate limit of movement is almost the same.

Furthermore, this process could be repeated when KX was replaced by other halide sources, such as NaX and ZnX_2_, as demonstrated in Figures S4 and S5. The change in the PL spectra of the samples that were synthesized by Cs_4_PbBr_6_ transformation with ZnX_2_ solutions, as shown in Figure S4a–b, suggested the Cs_4_PbBr_6_ successfully transformed to CsPbX_3_ in these solutions and realized the spectral shift. For different metal halides, the reaction time of interfacial anion exchange reaction and the wavelength range for the PL emission spectra are different. This phenomenon is thought to be caused by the influence of different metal cations on the diffusion of halogen ions at the interface.

**Figure 5:**
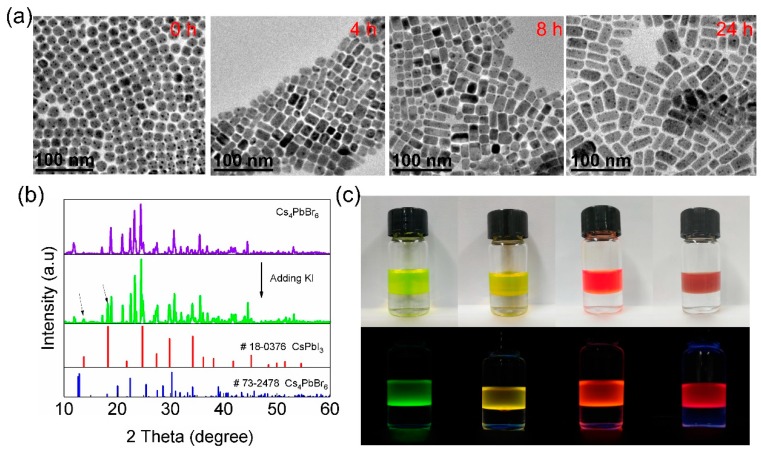
(**a**) Time-dependent TEM image of the product obtained by Cs_4_PbBr_6_ NCs reacting with KI aqueous solution. (**b**) XRD pattern of the final product of KI aqueous solution reaction. (**c**) Digital photos of the system under sunlight and UV light.

To detailed explore the process of interfacial anion exchange, TEM and XRD were applied to monitor the change on the morphology and size of typical CsPbBr_x_I_3−x_ NCs. Figure 5a shows the representative TEM images of Cs_4_PbBr_6_ NCs after 0, 4, 8, and 24 h when KI was added, respectively. At 4 h, the TEM image shows a mainly regular cuboid shape with a small portion of diamond-like shape, indicating the interfacial anion exchange reaction occurred. As storage time was extended, well-defined, and mono-dispersed, CsPbI_3_ NCs were achieved and their size increased significantly. The XRD pattern of the samples is depicted in Figure 5b. The diffraction peaks of the Cs_4_PbBr_6_ NCs were indexed as rhombohedral phase (JCPDS card No. 73-2478), and no other peaks were observed. When the original Cs_4_PbBr_6_ n-hexane reacted with the KI aqueous solution for only 4 h, main diffraction peaks at 2θ = 13.1°, 17.6°, 25.7°, 27.4°, 30.4°, 35.3°, 37.7°, and 44.4° were detected, indicating that the CsPbBr_x_I_3−x_ NCs were formed. Significantly, the peaks at 2θ = 15.2°, 21.6°, 26.5°, 30.6°, 34.4°, 37.8°, and 43.9° were observed, indicating that CsPbBr_3_ NCs were synthesized. Upon the addition of KI solution, due to the high solubility of CsBr in water solution (1243 g/L at 25 °C) [33], the decomposition of Cs_4_PbBr_6_ and the formation of CsPbBr_3_ NCs was driven. This is consistent with the phenomenon where the mixture turned green immediately after the KX solution was added into the Cs_4_PbBr_6_ n-hexane solution. Moreover, it is worth mentioning that no broad peaks at 2θ = 20–25° (a characteristic feature of amorphous materials) have been detected, which can be ascribed to the high crystallinity of Cs_4_PbBr_6_ and CsPbBr_3_ NCs. These results confirmed that at the KX aqueous solution interface, the driving force of water will first convert Cs_4_PbBr_6_ into CsPbBr_3_, while the halogen ion does not directly react with Cs_4_PbBr_6_ but with CsPbBr_3_.

The corresponding digital photos of four typical samples are shown in Figure 5c. When KI was added, the original transparent Cs_4_PbBr_6_ n-hexane solution divided into two layers immediately and showed a light green-yellow color in the top layer. With the time prolonged, the green-yellow color became yellow and red gradually, implying the interfacial anion exchange reaction between Br^−^ and I^−^ occurred. When the as-prepared products were irradiated with UV light, a bright photoluminescence was observed. Significantly, as the anion exchange reaction completed, the color of the sample synthesized by this method showed a much higher stability. After being stored for 48 h, the sample underwent UV light irradiation, the red solution kept almost the same color and no clear decay in photoluminescence was detected, implying high stability against moisture.

**Figure 6:**
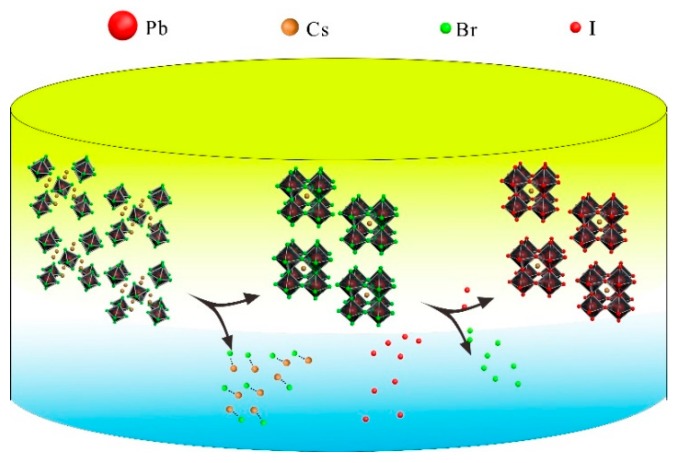
Illustration of the mechanism for the synthesis of CsPbX_3_ perovskite NCs by interfacial anion exchange reaction.

Through the above-mentioned analysis, a possible mechanism was proposed, as illustrated in Figure 6. The original material Cs_4_PbBr_6_ NCs were dispersed in n-hexane and their surface coated with the oleic acid and oleylamine, which resulted in hydrophobicity to ensure the stable presence of NCs in non-polar solvents. After adding KX aqueous solution, CsBr was stripped through the n-hexane/water interface due to its high solubility and the non-fluorescent Cs_4_PbBr_6_ NCs were converted to green fluorescent CsPbBr_3_ NCs. Meanwhile, oleic acid and oleylamine on the NCs surface are highly dynamic due to the water-driven mechanism, which makes the conversion process more accessible. It is well-known that KX has very low solubility in n-hexane but its solubility in water is very high and it would be quickly ionized into halogen ions in water. Therefore, the halogen anions in the aqueous phase have an excellent diffusion capacity at the n-hexane/water interface. When KX solution was added to Cs_4_PbBr_6_ NCs n-hexane solution, ionized halogen ions slowly diffused into the n-hexane and reacted with as-synthesized CsPbBr_3_ NCs causing the optical spectrum redshift or blueshift. Since the halogen ions diffusing near the n-hexane interface are consumed after the reaction, the diffusion behavior of the anions can be continuously performed without external energy input. Notably, the solubility of KX in n-hexane is extremely low; thus, only a small part of the K^+^ ion in the aqueous phase can enter n-hexane. This ensures the “pure” in that the anion exchange process does not create other non-luminescent substances. Additionally, when this system encounters slight stirring after adding KX aqueous solution, the rate of interface anion exchange can be accelerated.

**Figure 7:**
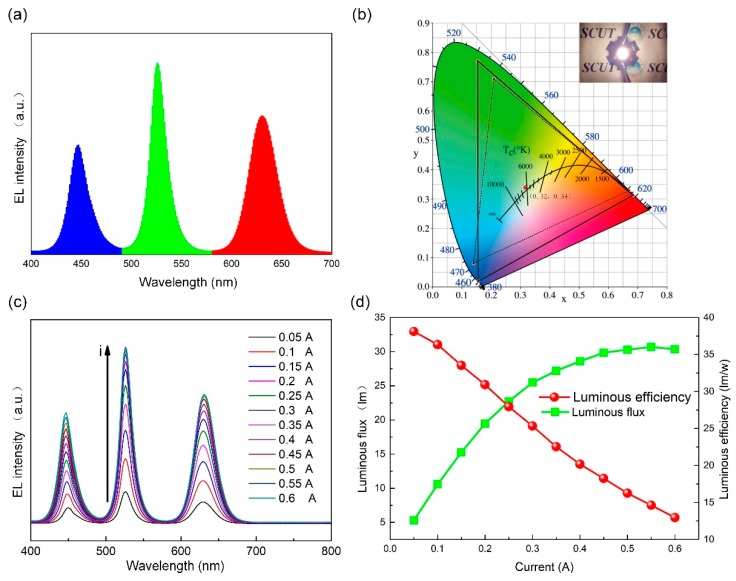
(**a**) Electroluminescence (EL) spectra of the white LED (WLED) device based on transformed CsPbBr_3_ NCs. (**b**) The International Commission on Illumination (CIE)t color diagram of the WLED device and the color gamut of blue LED, green CsPbBr_3_ NCs, red CdSe NCs (solid line), and NTSC standard (dashed line). The labeled red point in the c is the color coordinate of the CsPbBr_3_-based device. Inset shows a photograph of the device operating at a forward current of 100 mA. (**c**) Current-dependent EL spectra. (**d**) Luminous flux and luminous efficiency of WLEDs at different forward currents.

Perovskite NCs or other quantum dots are generally unstable at high temperatures [34]. Considering such thermal instability of the perovskite NCs and the heat generated by blue LED chips, an effective approach to avoid the temperature effect is to use the remote phosphor configuration, in which the phosphor layer is separated from the LED excitation light source [35,36]. To evaluate the as-synthesized CsPbX_3_ NCs for white LED (WLED) applications, the WLEDs, including green CsPbBr_3_ NCs film, a red CdSe film, and a blue LED chip, were fabricated. The electroluminescence (EL) spectra of Figure 7a revealed that the CsPbBr_3_ NCs have a narrow EL bandwidth, which coincides with the white light requirement of backlight displays. The International Commission on Illumination (CIE) chromaticity coordinates in Figure 7b shows that the WLEDs device presents cold white light (CCT is at approximately 6,300 and (x, y) is at around (0.32, 0.34), respectively) at different currents. Moreover, the WLEDs device shows an encompassing 125% color gamut of the National Television System Committee (NTSC, 1913) standard. This result indicates that the device could be useful as a next-generation candidate for high-end professional displays. The EL spectra of the WLEDs at different driving currents are shown in Figure 7c. The spectral intensity increased as current from 50 to 600 mA and the spectral shape remained almost unchanged, indicating that the CsPbBr_3_ NCs were not saturated by blue light. The luminous efficiency and flux of the WLEDs were determined, as shown in Figure 7d. The highest luminous efficiency was 32.7 lm/W, which is much higher than most perovskite quantum dot LEDs (Table S1).

## 4. Conclusions

In summary, we reported a facile and environmentally friendly method for the synthesis of highly stable and photoluminescent CsPbX_3_ through interfacial anion exchange reaction. By adding KX aqueous solution, we enabled the efficient chemical transformation of non-luminescent Cs_4_PbBr_6_ NCs to highly photoluminescent CsPbX_3_ NCs. The spectral shift of CsPbX_3_ NCs under different metal halide concentrations and sources were also investigated. By using different metal halides, the bandgap energy and PL spectra of CsPbX_3_ NCs were readily tuned over the entire visible spectral region. In addition, a chemical transformation mechanism between Cs_4_PbBr_6_ NCs and CsPbX_3_ NCs was investigated. The mechanism revealed that Cs_4_PbBr_6_ NCs dispersed in n-hexane were converted to CsPbBr_3_ NCs first by stripping CsBr through an interfacial reaction with aqueous solution. This process takes advantage of the high solubility of CsBr in KX aqueous solution, producing air-stable CsPbBr_3_ NCs, and then the CsPbBr_3_ NCs exchange with halogen ions at the interface of n-hexane/water. The interfacial anion exchange method eliminates the dependence of halogen ionic solubility on oleic acid and oleylamine. Moreover, water and n-hexane separated perovskite NCs from halogenated inorganic salts at the interface, so that non-luminescent substances could not enter into the CsPbX_3_ NCs in large quantities, ensuring their purity. WLEDs device were fabricated by combining the green luminescent CsPbBr_3_ NCs with red luminescent CdSe NCs and a blue GaN LED chip, obtaining a high color gamut of 125% (NTSC standard). There is still a need to obtain pure and highly stable CsPbX_3_ NCs, especially for CsPbCl_3_ NCs.

## Supplementary Materials

The following are available online at https://www.mdpi.com/2079-4991/9/9/1296/s1, Figure S1a,b. The statistics of width and length of the samples when KCl was added. Figure S1c,d. The statistics of width and length of the samples when KBr was added. Figure S1e,f. The statistics of width and length of the samples when KI was added. Figure S2: Element mapping of the samples prepared by Cs_4_PbBr_6_ transformation with KCl aqueous solution, Figure S3: XRD patterns of the samples prepared by Cs_4_PbBr_6_ transformation with (a) KBr and (b) KI aqueous solution, Figure S4: The PL spectra of the samples that were synthesized by Cs_4_PbBr_6_ transformation with different halide solutions (a) ZnCl_2_, (b) ZnI_2_, (c) NaCl, and (d) NaI, Figure S5. The color change of the samples that were synthesized by the Cs_4_PbBr_6_ transformation with different concentrations of (a) ZnCl_2_ and (b) ZnI_2_ aqueous solution. Table S1. The luminous efficiency of different types of perovskite light emitting-diodes.
