# Supplementary material for: Synthesis of Highly Photoluminescent All-Inorganic CsPbX3 Nanocrystals via Interfacial Anion Exchange Reactions
Source: Nanomaterials (Basel). 2019 Sep 11;9(9):1296. doi: 10.3390/nano9091296 (PMC6781013; doi:10.3390/nano9091296)
Supplement: Supplementary file 1 [file nanomaterials-09-01296-s001.pdf]

Supporting information

Synthesis of Highly Photoluminescent All-Inorganic $CsPbX_3$ Nanocrystals via Interfacial Anion Exchange Reactions

Zongtao Li[1], Cunjiang Song[1], Longshi Rao[1, 2] *, Hanguang Lu[1], Caiman Yan[1], Kai Cao[1], Xinrui Ding[1], Binhai Yu[1], and Yong Tang[1]

[1] Engineering Research Centre of Green Manufacturing for Energy-Saving and New-Energy Technology, School of Mechanical and Automotive Engineering, South China University of Technology, Guangzhou 510640, China

[2] College of Engineering, Shantou University, Shantou 515063, PR China

*Correspondence: memerls@mail.scut.edu.cn

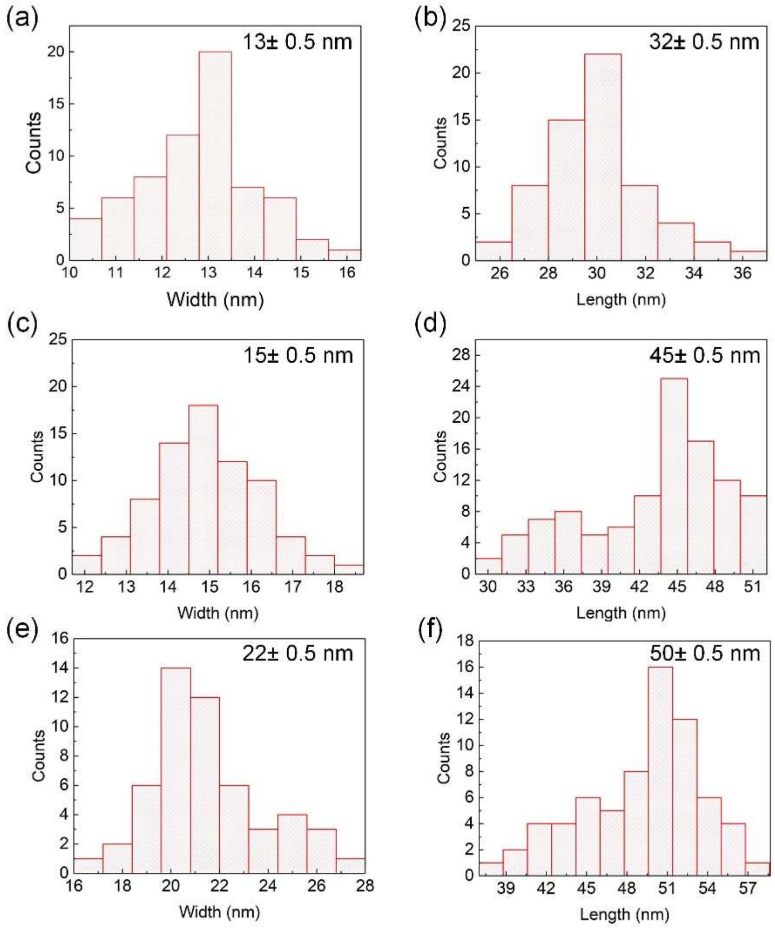

Figure S1. (a)-(b) The statistics of width and length of the samples when KCl was added. (c)-(d) The statistics of width and length of the samples when KBr was added. (e)-(f) The statistics of width and length of the samples when KI was added.

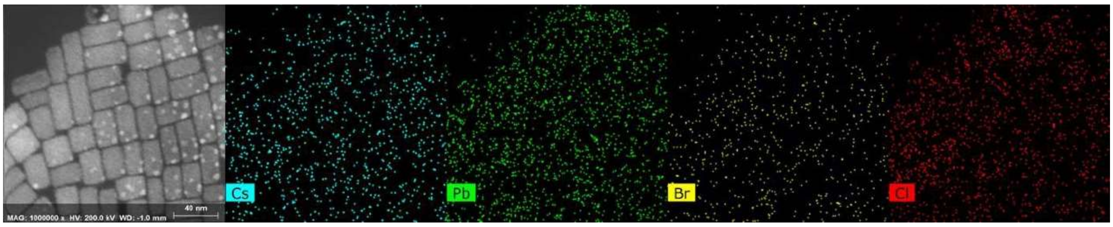

Figure S2. Element mapping of the samples prepared by Cs_4PbBr_6 transformation with KCl aqueous solution.

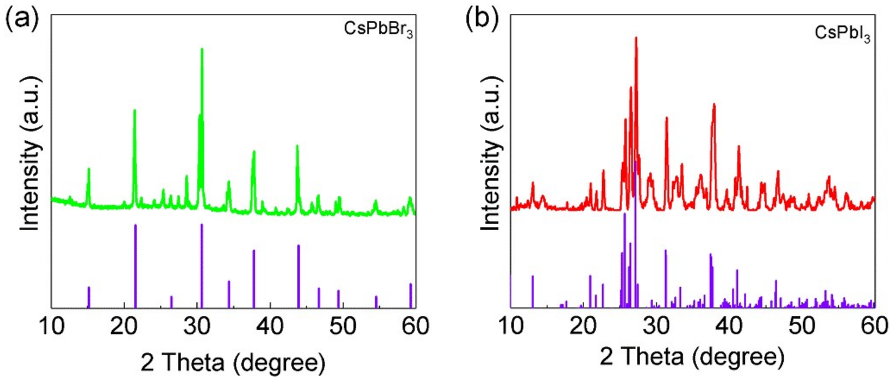

Figure S3. XRD patterns of the samples prepared by Cs_4PbBr_6 transformation with (a) KBr and (b) KI aqueous solution.

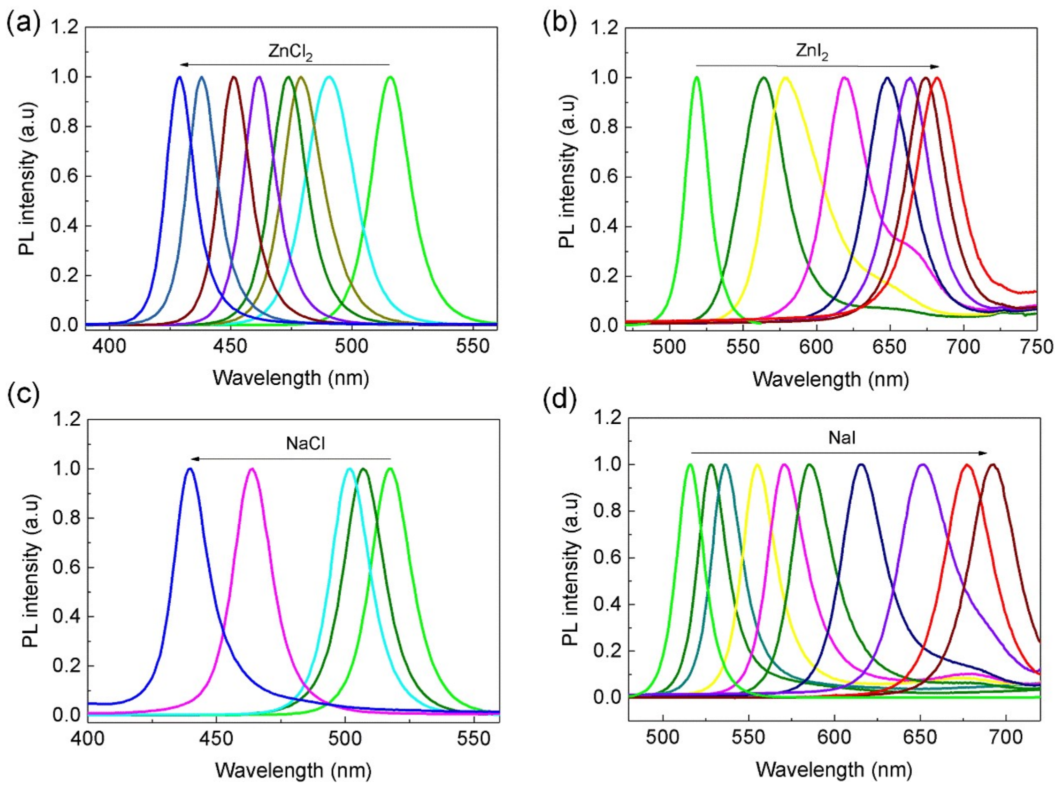

Figure S4. The PL spectra of the samples that were synthesized by Cs_4PbBr_6 transformation with different halide solutions (a) $ZnCl_2$, (b) ZnI_2, (c) NaCl, and (d) NaI.

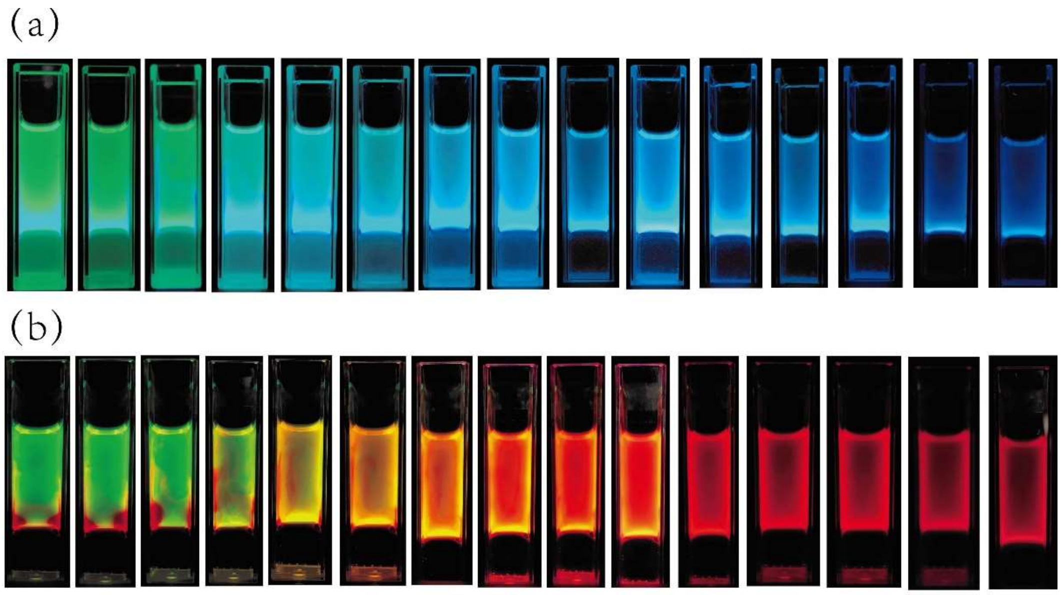

Figure S5. The colour change of the samples that were synthesized by the Cs_4PbBr_6 transformation with different concentrations of (a) $ZnCl_2$ and (b) ZnI_2 aqueous solution.

Table S1. The luminous efficiency of different types of perovskite light emitting-diodes

Type	Luminous efficiency (lm/W)	References
$CsPb_{1-x}Sn_xBr_3$ QD-LED	6.76	1
POSS-$CsPbBr_3$ NCs LED	14.1	2
$CsPb(Br_{0.3}I_{0.7})_3$ QDs-LED	19	3
MA-POSS $CsPbBr_3$ NCs LED	26.3	4
$CsPbBr_3$ QDs-LED	27.8	5
$CsPbBr_3$ QD-LED	30	6
$CsPbBr_3$ QD-LED	31.92	7
Ce^{3+}/Mn^{2+}-doped $CsPbBr_3$ QD-LED	42	8
$CH_3NH_3PbBr_3$ QD-LED	48	9
$CsPbBr_3$ NCs LED	32.7	This work

References:

[1] Wang H C, Wang W, Tang A C, et al. High-Performance $CsPb_{1-x}Sn_xBr_3$ Perovskite Quantum Dots for Light-Emitting Diodes[J]. Angewandte Chemie International Edition, 2017, 56(44): 13650-13654.

[2] Huang H, Chen B, Wang Z, et al. Water resistant $CsPbX_3$ nanocrystals coated with polyhedral oligomeric silsesquioxane and their use as solid state luminophores in all-perovskite white light-emitting devices[J]. Chemical science, 2016, 7(9): 5699-5703.

[3] Singh B P, Lin S Y, Wang H C, et al. Inorganic red perovskite quantum dot integrated blue chip: a promising candidate for high color-rendering in w-LEDs[J]. RSC Advances, 2016, 6(83): 79410-79414.

[4] Pan A, Wang J, Jurow M J, et al. General strategy for the preparation of stable luminous nanocomposite inks using chemically addressable CsPbX3 peroskite nanocrystals[J]. Chemistry of Materials, 2018, 30(8): 2771-2780.

[5] Ren J, Dong X, Zhang G, et al. Air-stable and water-resistant all-inorganic perovskite quantum dot films for white-light-emitting applications[J]. New Journal of Chemistry, 2017, 41(22): 13961-13967.

[6] Wang H C, Lin S Y, Tang A C, et al. Mesoporous Silica Particles Integrated with All-Inorganic $CsPbBr_3$ Perovskite Quantum-Dot Nanocomposites (MP-PQDs) with High Stability and Wide Color Gamut Used for Backlight Display[J]. Angewandte Chemie International Edition, 2016, 55(28): 7924-7929.

[7] Li C, Zang Z, Chen W, et al. Highly pure green light emission of perovskite $CsPbBr_3$ quantum dots and their application for green light-emitting diodes[J]. Optics express, 2016, 24(13): 15071-15078.

[8] Pan G, Bai X, Xu W, et al. Impurity ions codoped cesium lead halide perovskite nanocrystals with

bright white light emission toward ultraviolet–white light-emitting diode[J]. ACS applied materials & interfaces, 2018, 10(45): 39040-39048.

[9] Zhang F, Zhong H, Chen C, et al. Brightly luminescent and color-tunable colloidal $CH_3NH_3PbX_3$ (X= Br, I, Cl) quantum dots: potential alternatives for display technology[J]. ACS Nano, 2015, 9(4): 4533-4542.
